# Creating a locally driven research agenda for the ethnic minorities of Eastern Myanmar

**DOI:** 10.1186/s12961-019-0465-7

**Published:** 2019-06-26

**Authors:** Eva Purkey, Saw Nay Htoo, Rachel Whelan, Naw Pue Pue Mhote, Colleen M. Davison

**Affiliations:** 1 Department of Family Medicine, Queen’s University, 220 Bagot street, Kingston, Ontario K7L 3G2 Canada; 2 Burma Medical Association, PO Box 156, Mae Sot, Tak, 63110 Thailand; 3 Community Partners International, 81 University Avenue Road, Shwe Taung Gyar Ward (1), Bahan Township, Yangon, Myanmar; 4 Department of Public Health Sciences, Faculty of Health Sciences, Queen’s University, Carruthers Hall, 62 Fifth Field Company Lane, Kingston, Ontario K7L 3N6 Canada

## Abstract

**Background:**

Research funding and production is inequitably distributed internationally, with emphasis placed on the priorities of funders and international partners. Research capacity development, along with agenda-setting for research priorities can create agency and self-sufficiency and should be inclusive of all relevant stakeholders. Myanmar is a fragile state, where decades of conflict have created a weakened healthcare system and health research sector. The population of Eastern Myanmar have long had their healthcare needs met by community-based organisations and ethnic health organisations operating within Eastern Myanmar and the adjoining Thai–Myanmar border. Despite a transition to civilian rule, the current context does not allow for a truly participatory health research capacity development and agenda-setting exercise between the health leaders of Eastern Myanmar and the government in Yangon. In this context, and with a desire to enhance the capacity, legitimacy and agency of their organisations, the health leaders of Eastern Myanmar are seeking to develop their own health research capacity and to take control of their own research agenda.

**Methods:**

Approximately 60 participants from 15 organisations attended a 3-day forum with the goals of (1) developing research capacity and interest through a research conference and methods workshop; (2) using a nominal group technique (NGT) to develop a locally driven research agenda; and (3) supporting the development of local research projects through ongoing funding and mentorship.

**Results:**

Participants were actively engaged in the workshops and NGT. Participants identified a broad range of health issues as priorities and were able to develop consensus around a list of 15 top priorities for the populations they serve. Despite availability of ongoing support, participants did not pursue the opportunity to engage in their own research projects emerging from this forum.

**Conclusions:**

The NGT was an effective way to achieve engagement and consensus around research priorities between a group of healthcare providers, researchers and policy-makers from a variety of ethnic groups. More active involvement of senior leadership must happen before the energy harnessed at such a forum can be implemented in ongoing research capacity development.

## Background

Research funding is inequitably distributed both geographically and with respect to health priorities, and often fails to address the most pressing needs of the global population [1]. The term 10/90 gap was developed in the 1990s to refer to the phenomenon by which only 10% of global funding for research is spent on diseases affecting 90% of the world’s population [2]. While the nature of this gap has changed in the intervening two and a half decades, with increased overall funding for global health research, improvements in innovation, and an increasing number and variety of actors, the gap itself nevertheless remains present today [1]. The 10/90 gap exists largely because research priorities, even those addressing concerns within low- and middle-income countries (LMICs), are often set by funders, international partners and organisations, rather than by the communities who will bear the consequences of what research is or is not prioritised [3]. Whether a cause or a consequence of this, only a very small proportion of research publications emanate from LMICs. For example, despite the fact that 75% of the global burden of cardiovascular disease is found in LMICs, a random selection of 3000 citations found that only 6–8% of peer-reviewed articles were from these countries [4].

The 1990 Commission on Health Research and Development laid out four priorities for health research, namely (1) that all countries should undertake essential national health research, including the establishment of national priorities and the development of local research capacity; (2) that LMIC (then called developing countries) research should be supported by international partnerships; (3) that financial support from international sources should be mobilised to support the research needs of LMICs; and (4) that there should be an international monitoring system for ensuring progress, and for promoting financial and technical support for the research addressing health problems of LMICs [5].

Over the intervening three decades, many countries have worked on priority-setting initiatives [6]; however, there remain challenges in developing participatory processes involving all relevant stakeholders. This has been even more challenging in fragile state contexts where certain ethnic groups, for example, are either in active armed conflict with the national government or have a history of experiencing overt discrimination or neglect [7, 8].

Participatory research is increasingly recognised as an important tool for translating research knowledge into action [9] as opposed to having it sit in the academy or in broad legislative frameworks with little impact on the populations served. Additionally, participatory research can be used as a tool for self-determination and for social justice [9]. This is particularly relevant in contexts such as that of Eastern Myanmar where populations long marginalised wish to have control over research, knowledge creation and programmes that affect them [9]. Finally, participatory research can ensure that research is culturally and logistically appropriate, creates sustainable systems and programmes, and supports capacity development in stakeholders among others. [10]

**Fig. 1:**
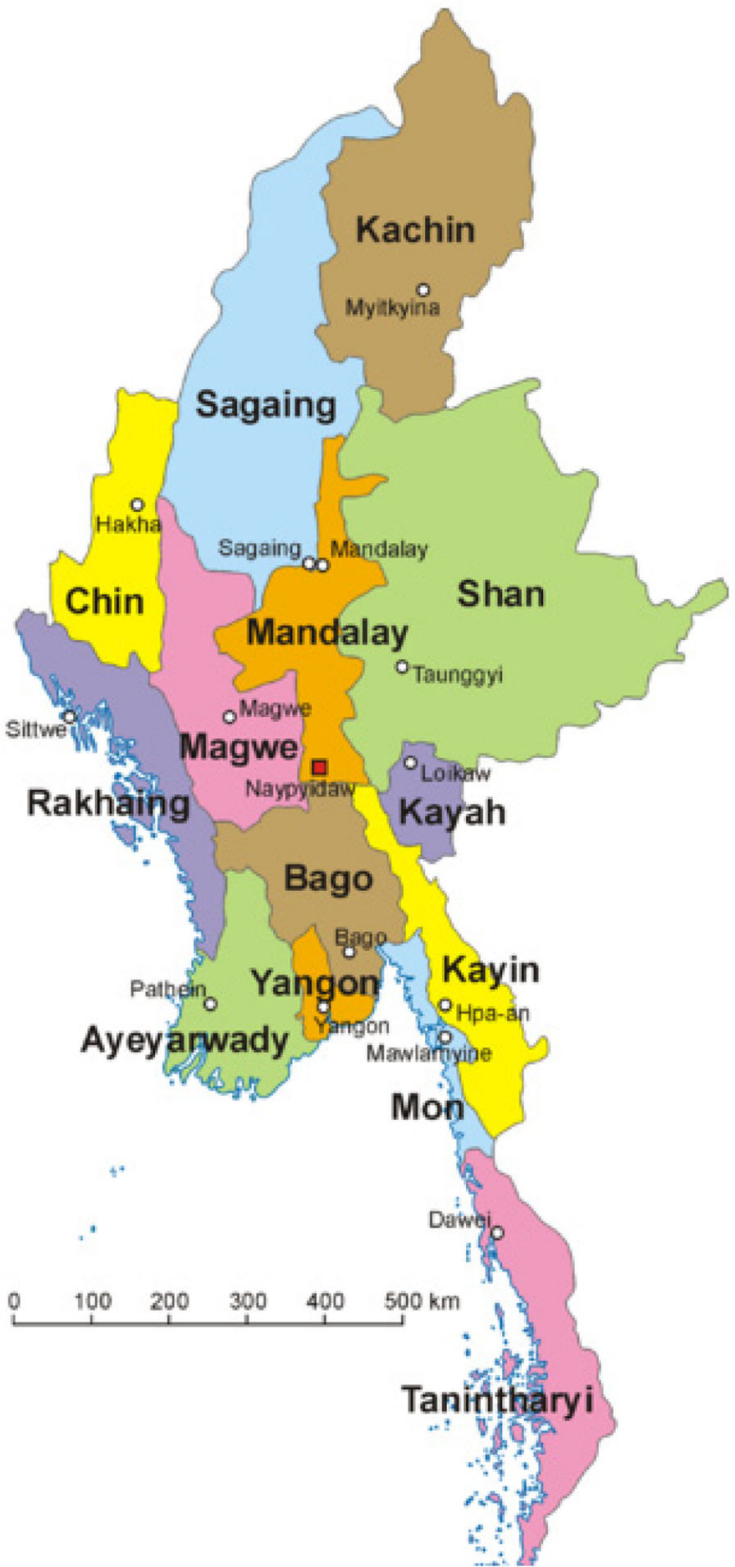
Map of Myanmar (courtesy of https://en.wikipedia.org/wiki/Myanmar)

Myanmar is a nation in which decades of conflict, political repression and mismanagement have resulted in a very weakened healthcare system [11–13]. While there have been some improvements since 2010 with changes to the political system [12], significant challenges remain, particularly in remote and ethnic areas of the country. Myanmar is also a country suffering from a double burden of communicable and non-communicable diseases (NCDs), in which 59% of total mortality is estimated to come from NCDs, 30% from communicable and maternal/perinatal conditions, and 11% from injuries and accidents in the country as a whole [14]. While the Myanmar government has identified many important priorities relevant to Eastern Myanmar in its 2017–2021 National Health Plan [15], including collaboration with ethnic health organisations (EHOs), task shifting, support for health information systems and the promotion of health research, there is great distrust between the EHO and community-based organisations (CBOs) of Eastern Myanmar with regards to respectful, participatory implementation of these recommendations. Eastern Myanmar encompasses Shan state, Kayah state, Kayin state, Mon state and Tanintharyi division (Fig. 1), and is home to many different ethnic minorities, including Shan, Karenni, Karen, Mon and Pa-O, among others. Some of these groups are still in active conflict with the military, while others have been displaced or have had their human rights threatened by various extractive industries and development activities [16–18], all of which have an impact on health and healthcare delivery. These ethnic minorities of Eastern Myanmar represent a distinct subset of the population and previous data suggests that, while they too suffer from a double burden of disease, their rates of communicable diseases related to inadequate access to water and sanitation, persistent presence of malaria and inadequate access to reproductive health services, are higher than the country’s average [19, 20].

EHOs and CBOs have been providing healthcare to the migrant and ethnic populations in Eastern Myanmar and on the Thai–Myanmar border for decades [17]. While their focus has been on the provision of clinical care and public health interventions through stationary and mobile clinics, in 2002, several of these organisations came together to form the Health Information Systems Working Group (HISWG), which seeks to strengthen the healthcare system in Eastern Myanmar with a focus on data management and information sharing [21]. As part of their work, the EHOs and HISWG have been collecting data and doing research with partners for many years (Table 1). Partnerships have developed with international academic institutions as well as non-governmental organisations for this work.

**Table 1:** Sample of recent and ongoing research projects in Eastern Myanmar and the Border region involving ethnic health organisations and community-based organisations

Title of Project	Partners	Funders	Outputs/Results
Reproductive health assessment in the eastern border region of Burma	World Refugee Council, Community Partners International, Burma Medical Association, Back Pack Health Workers Team, Karen Department of Health and Welfare, Mae Tao Clinic	MacArthur Foundation and Swiss Agency for Development and Cooperation	Explored issues around access to sexual and reproductive health services, including cost, decision-making, pregnancy services and place of delivery, family planning, supply chain, and impact of security threats on access to sexual and reproductive health
Health and human rights in Eastern Myanmar after the political transition: a population-based assessment using multistaged household cluster sampling	Harvard Medical School, Community Partners International, University of Washington, Burma Medical Association, Health Information Systems Working Group, Back Pack Health Worker Team, Mae Tao Clinic, Karen Department of Health and Welfare, Karenni Mobile Health Committee, Mon National Health Committee, Harvard School of Public Health, University of California	Burma Relief Centre	Household surveys in eastern Myanmar from July 2013–September 2013 exploring demographics, mortality, health outcomes, water and sanitation, food security and nutrition, malaria, and human rights violations
Human resources for health: task shifting to promote basic health service delivery among internally displaced people in ethnic health programme service areas in eastern Burma/Myanmar	Community Partners International Burma Medical Association, Health Information System Working Group, Back Pack Health Worker Team, Mae Tao Clinic, Karen Department of Health and Welfare, Department of Global Health and Development London School of Hygiene and Tropical Medicine	Department of Foreign Affairs and Trade (Canada), Burma Relief Center, United Kingdom Department for International Development through Christian Aid, United States Agency of International Development through the International Rescue Committee	Exploration of the effective healthcare service delivery by ethnic health organisations and community-based organisations through standardised training and task shifting in the context of a shortage of formally trained skilled medical professionals
Impact of community-based maternal health workers on coverage of essential maternal health interventions among internally displaced communities in Eastern Burma: The MOM Project	Center for Public Health and Human Rights, Bloomberg School of Public Health, UCLA School of Medicine, Global Health Access Program, Burma Medical Association, Karen Department of Health and Welfare, Mae Tao Clinic	Bill and Melinda Gates Institute for Population and Reproductive Health at the Johns Hopkins Bloomberg School of Public Health and the Foundation for the People of Burma. Additional funds for the final survey were provided by a gift to the Center for Public Health and Human Rights at Johns Hopkins	Outcome evaluation of MOM project (frequency of antenatal care, postnatal care, contraceptive use, unmet need for contraception, skilled birth attendants, etc.)
Community-based delivery of maternal care in conflict-affected areas of eastern Burma: perspectives from lay maternal health workers	Center for Public Health and Human Rights, Johns Hopkins Bloomberg School of Public Health, Global Health Access Program Berkeley, Burma Medical Association	Bill and Melinda Gates Foundation	Community-based workers’ perspective on rights-based approach to service delivery in a conflict setting in the context of widespread human rights violations
Health and human rights in Karen State, Eastern Myanmar	Center for Public Health and Human Rights, Bloomberg School of Public Health, Karen Department of Health and Welfare, Division of General Internal Medicine and Health Services Research, University of California Los Angeles Community Partners International, Physicians for Human Rights, University of Minnesota Medical School, Human Rights Center, University of California, Berkeley	Open Society Institute, the National Endowment for Democracy, and the Oak Foundation	Cluster survey of households in eastern Myanmar assessing health status, access to healthcare, food security, exposure to human rights violations and identification of alleged perpetrators over the 12 months prior to January 2012

While the Commission on Health Research and Development’s recommendations would suggest that the Myanmar government should be responsible for engaging ethnic stakeholders in research priority development, the long history of conflict between these groups makes that challenging [8]. Anticipating major changes to the national healthcare system following the democratic elections of 2010 and 2015, a group of EHOs developed a plan for eventual convergence between the ethnic health systems and the national health system. However, in the same way that setting a research agenda creates agency for a national government, HISWG and the EHOs are interested in having more control over their own research and research agenda, developed in response to their perceived needs, independent from national government priorities but also independent from, and able to influence, the priorities of foreign donors and academic institutions.

In this context, a partnership between the Burma Medical Association (BMA, one of the lead organisations in HISWG), Queen’s University in Kingston, Canada, and Community Partners International (an American NGO based in Yangon, Myanmar) was formed to start working on health research capacity-building and health research agenda-setting for Eastern Myanmar and the Thai–Myanmar border area through an inclusive, participatory research methods and priority-setting forum.

### Processes

Nuyens proposes that ‘processes’ are the mechanisms by which stakeholders are involved and decide on research priorities [6]. Limitations to evidence-based agenda-setting can include limitations in the availability of quality data about populations in question and this limitation can be particularly obvious when looking at marginalised groups or subsets of a national population to which national findings might not apply. Survey data, publicly available reports [19, 20], as well as some peer-reviewed publications [22, 23] outlining health conditions and epidemiology are available on the populations of Eastern Myanmar, particularly from the work of HISWG.

Several agenda-setting methodologies are outlined in the academic literature [24]. Comprehensive approaches employing specific tools have been developed by the Child Health and Nutrition Research Initiative (CHNRI), the Council on Health Research and Development (COHRED) and others. The nominal group technique (NGT) is a method in which consensus is achieved through discussion and iterative secret ballot. Developed in the United States in the 1960s, this technique usually involves a small group [9–12] of experts who provide information, rate, discuss and re-rate a series of items or priorities by secret ballot [25]. NGT has been used previously in priority-setting exercises related to physiotherapy research, general practice, stakeholder consensus and implementation science [26–29]. Challenges in using NGT include how to determine which ‘experts’ to include, as well as how to assess the accuracy of the answers obtained, since consensus does not necessarily imply objective truth [30]. Advantages include a flattening of hierarchies as no one person’s view takes priority over others, and the use of secret ballot may allow people more freedom in articulating their opinions [30]. The NGT protocol includes approximately 5 steps per ‘round’ (Table 2). Following introductions and explanations, several rounds can be used in succession to develop consensus on a topic, in this case, the research priorities for Eastern Myanmar.

**Table 2:** Nominal Group Technique protocol

1. Silent generation of ideas: In this step, participants used sticky notes to write down as many research priorities as they could think of, on their own, without consulting their peers	
2. Sharing of ideas: each participant presented their ideas and put their sticky notes up on a shared board	
3. Group discussion and grouping of ideas: participants, as a group, discussed the ideas presented. No ideas were eliminated, but some ideas were grouped into common themes or groupings. For ideas to be grouped, the participant who generated the idea needed to agree that the idea was the same as the others in the group	
4. Voting and ranking: after discussion participants voted individually, in silence	
5. Compilation of votes and feedback: votes were compiled, and priority ranking lists were fed back to participants. At this point, steps 3 through 5 restart as the priority lists often generate new ideas and points of discussion for participants, causing some participants to change their rank order. Steps 3–5 were repeated twice (secret ballot 1 and 2) in small groups and twice (secret ballot 3 and 4) in the large group	

### Objectives

The objectives of the health research agenda-setting exercise were three-fold. The primary objective was to develop a research agenda with local stakeholders for Eastern Myanmar and the Thai–Myanmar border region which could be used to direct international partnership in research as well as local research activities. A second objective was to highlight some of the excellent research work already being done in Eastern Myanmar and the border region through group discussions throughout the process. The third objective was to increase interest and engagement in the idea of research among EHOs and CBOs in order to enable participants to be actively involved in meeting the goals of their own research agenda.

Throughout this process, our explicit priority was to be led by the goals and preferred methods of our local co-investigators from the BMA, ensuring that from conceptualisation to completion, this was a community-led project enhancing local self-determination and community capacity development.

## Methods

Local co-investigators from the BMA recruited participants from 16 organisations (Table 3), representing predominantly Mon, Shan, Karen, Karenni and Pa-O ethnic groups. These organisations have been involved in HISWG and other health system strengthening initiatives and represent the main ethnic groups in Eastern Myanmar. Individual participants were identified by their organisation. This was an intentional sampling by participant organisations, but was not necessarily representative of populations served, with an over-representation of participants from organisations in proximity to the forum site due to cost and logistics of travel. Attendance varied only slightly from day to day, and participants in Table 3 are those who were present on the final day, the day of the NGT.

**Table 3:** Participant number by organisation (April 11th)

Organisation	Number of participants on April 11th
Burma Based Health Services-Mae Tao Clinic	1
Burma Medical Association	20
Back Pack Health Workers Team	6
Children Development Center	1
Civil Health and Development Network (Karenni State)	4
Democratic Karen Benevolent Army	2
Karen Department of Health and Welfare	5
Karen Human Rights Group	2
Khon Kaen University	1
Malaria Elimination Task Force	1
Mon National Health Committee	2
Mae Tao Clinic	7
Pa-Oh Health Working Committee	2
Thammasat University (School of Global Studies)	1
Shan State Development Foundation	1

Curriculum was developed in collaboration by Queen’s University, BMA and Community Partners International leads. These three organisations acted as co-hosts to the event, with contributions from local researchers and health system leaders. Following 2 days of orientation to the research process and in-depth discussions about existing local research activities, during which participants developed research questions and sampling strategies, considered available data sources, and reviewed research methodology and ethics, participants engaged in a brainstorming exercise to identify potential criteria by which they would rank research priorities. After discussion, criteria were elicited through secret ballot in which participants were invited to identify all criteria that seemed relevant to them. Criteria identified by participants are listed in Table 4.

**Table 4:** Criteria for prioritisation

• Number of people affected	
• Greatest danger for the community	
• Community priorities and ownership	
• Impact on the most vulnerable community members	
• Feasibility	
• Availability of funding	
• Human rights (rights of the child, women’s rights)	
• Political stability	

Day three was allocated entirely to a NGT [25, 26, 30]. Because of the use of secret ballots, as discussed above, the NGT has the potential to encourage participation by flattening some of the hierarchies that can exist within groups [30]. Given the large number of participants at this workshop, the group was divided into four sub-groups of approximately 15 people. These groups were stable over the course of the workshop, and while there was some mixing of organisations, there was a tendency among participants to stay together with members of their own organisation, or of their own language group, when numbers allowed. Each group completed two rounds of NGT (secret ballots 1 and 2), at which point their top five priorities were fed back to the larger group. The larger group then engaged in a dynamic discussion about the remaining 20 priorities, grouping and re-grouping them into a final 15 priorities. At that point, participants ranked these priorities using a secret ballot (secret ballot 3), which was followed by a final group discussion and a final secret ballot ranking (secret ballot 4). Table 2 outlines the step-by-step NGT process that was used.

As a final component, funding and logistical support were available to support a small research competition. Participants were invited to submit draft proposals based on the priorities identified during the forum over the following 4 weeks. Applicants would then be mentored through ethics submission to the Border-based Community Ethics Advisory Board, development of methods, implementation of research project, and knowledge translation of a small research project specifically related to NGT-identified health and health system priorities, hence further stimulating the development of local research capacity through a successful research experience.

Ethics approval was obtained by Queen’s University Health Sciences and Affiliated Teaching Hospitals Research Ethics Board.

## Results

### Results of the NGT process

Given the large number of participants, an extremely rich set of potential priorities was initially generated and inevitably some of the subtleties were lost in the process of coming to consensus. The initial brainstorm is retained here in Table 5 as it illustrates the diversity of challenges facing the ethnic populations of Eastern Myanmar and the border region, as well as how broadly participants defined ‘health’.

**Table 5:** Initial Nominal Group Technique brainstorm

Theme	Individual research topics
Infectious disease	• Tuberculosis • HIV • Worms • Diarrhoea • Malaria
EPI (Expanded Programme of Immunisation)	• Immunisation for under 5 • Immunisation for rabies, hepatitis • Immunisation coverage
Non-communicable diseases (NCDs)	• Diabetes mellitus • Heart problem • Stroke • Hypertension • Eye problems • Anaemia • Epilepsy • Disability • Extent of NCDs in ethnic areas • Medication adherence
STI/HIV	• STI health education to reduce prevalence of STIs • HIV/AIDS and availability of treatment
Family planning (FP)/reproductive health	• FP and cultural belief • Reproductive health/family planning • Early marriage • Teenage reproductive health • Breastfeeding
Antenatal care (ANC)/Safe delivery	• At least 4 ANC visits for every pregnancy • Anaemia • Safe delivery • Teenage pregnancy • Traditional birth attendants • Home delivery
Gender	• Gender based violence • Domestic workers • Early marriage
Mental health	• Mental health • Mental health and alcohol use among married men • Stress • Social and mental health among men who use injection drugs
Drug use	• Betel nut chewing • Smoking • Alcohol use • Drug use (particularly methamphetamines, yaba) • Drug use among youth
Accidents	• Accidents and injuries • Road traffic accidents • Helmet use • Landmine injuries • Disability
Water, sanitation and hygiene (WASH)	• Household waste management and disposal • Toilets and latrines • Safe water • Mercury levels in water • School health and hygiene • Handwashing
Food safety/security and nutrition	• Nutrition and lifestyle • Under five nutrition • Nutrition in pregnancy • Nutrition for the elderly • Relationship between nutrition and oral hygiene • Level of food safety knowledge among school children • Vitamin A and deworming • Under five obesity • Health impact of fertilisers • Food security • Food safety • Awareness of health food
Environmental and human-made disasters	• Negative consequences of deforestation on health • Replanting to prevent deforestation • Plants that can endanger the environment • Factory industries • Mining industries (deforestation, toxins in water, ecological impact) • Dam • Civil war and political conflict • Impact of agricultural practices on health • Rabies prevention, reduce number of stray dogs • Occupational health
Health service provision	• Access to healthcare in Eastern Burma • Access to primary healthcare services • General health screening • Private pharmacies in the community • Drug control (pharmaceuticals) • Community health workers training • Trained traditional birth attendants • Health worker training at all levels • Health system logistics and infrastructure • Community involvement in health sector • Transportation
Health education	• Health education on communicable diseases • Health education on non-communicable diseases • Nutrition education • Awareness around alcohol and drug use • Awareness of tuberculosis and malaria
Economic situation	• Household income • Occupation
Education	• Future concern among students in migrant learning centre • Educational opportunities for disabled children/youth • School-aged children without access to school
Social exclusion	• Child abuse • Abandoned child • Social exclusion
Migration	• Population mobilising • Migration • Myanmar migrants within Thailand
Others	• Political ideology • Cultural beliefs

After the first two rounds of NGT in small groups (secret ballot 1 and 2), 15 priorities were retained for consideration by the larger group (Table 6). Following extensive debate, participants elected to separate malnutrition into two populations (vulnerable and under-five) and to separate water issues into water, sanitation and hygiene (traditional WASH) and water quality, by which they meant water contamination through mining, etc. These 15 priorities were then ranked individually (secret ballot 3) by all 60 participants, a second discussion was had, and a final secret ballot (secret ballot 4) ranking was performed. The final priority list can be found in order in Table 6 and includes topics related to maternal and child health; mental health, alcohol and drug use; food security, water and sanitation; and NCDs, among others.

**Table 6:** Final research priorities identified, in order of ranking

1. Water quality and contamination (e.g. mining, etc.)	
2. Mental health	
3. Illegal drug use	
4. Under-five malnutrition	
5. Pharmaceuticals	
6. Antenatal care	
7. Food security	
8. Non-communicable diseases/hypertension	
9. Immunisations	
10. WASH	
11. Health education	
12. Family planning	
13. Alcohol consumption	
14. Safe delivery	
15. Malnutrition among vulnerable populations	

### Results of the research competition

The proposed research competition was not successful as envisioned in that no proposals were submitted despite outreach on the part of BMA and offers of support in proposal development.

## Discussion

### Priority-setting process

Despite the lack of research expertise and experience of some participants, together, participants had a wealth of knowledge and expertise concerning the health issues and priorities in their communities. This echoes back to the importance of participatory research, as this depth and breadth of local knowledge is virtually impossible to obtain without community engagement and involvement. Table 5 shows how broadly participants defined health, and how they perceived the challenges facing their communities to overlap between communicable, non-communicable and environmentally triggered conditions, some of which are uniquely related to ongoing conflict and militarisation [19].

The NGT process generated a large number of topics illustrating a variety of research and clinical areas of focus, which could be explored in more depth when developing research questions. Participants were able to discuss amongst themselves how to organise priorities for research in their communities, and all were clear on the importance of community input and oversight when identifying research projects and priorities.

Throughout the discussions, both during the NGT process and on day 2 of the workshop, when groups were brainstorming research questions, three items came up repeatedly for discussion, namely illegal drug use, NCDs and mental health.

Myanmar is one of the main producers of opium and methamphetamines in the world [31], and there was broad agreement among participants about the scourge this creates in small ethnic communities with high prevalence of drug use and addiction. Referring to their criteria for prioritisation from day 1 (Table 4), participants felt that while illegal drug use was retained as one of the top research priorities, in practice they would be unable to research or intervene on illicit drug use given security risks in a context where the market is dominated by potentially dangerous armed groups.

NCDs were another priority topic. It is unlikely that EHOs have the infrastructure or capacity, at this time, to screen for NCDs, and so the prioritisation of NCDs appears from participant discussion to come from increasing manifestations of end-stage disease such as cerebrovascular events, renal failure and complications of diabetes leading to various levels of disability. Information on prevalence of NCDs in Myanmar is limited [32, 33], and this is a relatively uncontroversial topic that could be an interesting, important and emerging area for research leading to implications for programme development, funding and clinical care.

Finally, mental health was discussed repeatedly. Men’s mental health was identified as a priority, as was the relationship between mental health, alcohol and other substance use. Mental health consequences of recurrent displacement, conflict and human rights abuses, but also more chronic consequences of poverty and marginalisation are all topics that warrant further investigation in this context.

Interestingly, throughout the process of four rounds of NGT, all formal health systems priorities were dropped from the final list. It is unclear why this happened, except that perhaps participants found specific health issues more compelling and failed to see in this context the system challenges that may underlie them. Importantly, EHOs have collected health systems data (predominantly unpublished clinic-level data such as availability of pharmaceuticals, number of staff, vaccination procedures, etc.) and further support in the analysis of this data may help participants to see the relationship between system-level challenges and ongoing health concerns. Health systems priorities would also likely re-emerge as individual research questions related to priority topics were developed.

### Research competition

Despite having funding and mentorship available, no participants took up the offer of submitting a research proposal, regardless of encouragement and outreach from organisers.

A paper by McGregor et al. [24], reviewing priority-setting initiatives in LMICs, found that 78% of initiatives provided no evidence of implementation or follow-up strategies. We explicitly did not wish to become part of this statistic, hence the plan for project support, as well as plans for a second forum to be held a year later. Nevertheless, this effort was not successful. This challenge may result from the potentially intimidating nature of the research process, which we were trying to mitigate through the offer of intensive mentorship. This offer of mentorship itself, particularly that offered by Western academics, may have been intimidating, and we sought to minimise this by ensuring that BMA was the main point of contact. This failure may also result in part from participant selection as discussed in limitations section below. As discussed above, despite active engagement during the forum, many participants would not normally have research as part of their job description. Hence, upon returning to their organisations, they might have had neither the time nor organisational support to engage in research activities. While some decision-makers and heads of organisations were present, most participants were mid-level and/or programme-level staff, and it might be necessary to have a greater presence of senior leadership in order for implementation to be successful. Presence of senior leadership might ensure that they were engaged in the process of identifying research as an organisational priority, which would be essential if they are then to act as support both in terms of encouragement but also in terms of prioritising ongoing research within their organisation from a job description and time perspective.

### Limitations

NGT is a recognised process for priority-setting in health research and was effectively used to actively engage all participants in this setting. Nevertheless, both the NGT process and the subsequent research competition were limited to some extent by two important factors. The first of these was the lack of population-specific data mentioned above. While participants would be familiar with the reports cited in this paper, as many of them were involved in data collection, the findings of these reports were not formally reviewed as a part of the process. Incorporating more time into the forum to review these documents could have resulted in more robust recommendations. In addition, given the paucity of data available on certain areas of health of the ethnic populations of Eastern Myanmar, the process might have benefitted from additional data sources such as project reports and community needs assessments, providing participants with a richer data set from which to consider their priorities. Local organisers felt that national data does not represent their specific communities, hence this was not considered to be a relevant source of information although might have provided a point for discussion.

The second limitation was that the participants were divided between a few who had significant research experience, several who had some, and many who had very little, often administrators or healthcare providers by training. Ensuring the participation of a variety of stakeholders representing research, policy-makers and healthcare providers with input and knowledge about local populations is best practice [6] when considering population health and health research needs, recognising that necessarily not all these stakeholders will have an equal understanding of the research process. In practice, however, participants struggled to distinguish between programming and clinical priorities, on the one hand, and research priorities on the other (with research priorities being areas in which more research is needed to better understand the problem or its solutions, whereas in the case of health priorities, there may be significant knowledge available even if the problem remains unresolved). Better preparation of participants prior to the meeting as well as more thorough review of available literature might have helped with this differentiation and might also have encouraged participants to consider developing their own research proposals.

## Conclusion and next steps

The CHNRI expert group identified 20 universal challenges in setting priorities, including ensuring that the process can be repeated and validated, and that it is iterative with a feedback loop [34]. We believe that this forum was a first step, and that more work is needed to ensure that there is stability among research priorities. Despite the lack of follow-up in terms of research projects, the reasons for which could be investigated further, participant EHOs are currently in the process of implementing a large household survey looking at health of their population. Likewise, this process has resulted in the development of international relationships that have led to research capacity development and specific projects outside of the context of this forum. Finally, there remain plans to hold a second research workshop in a different format, and the availability of recently collected data from the household survey will create an opportunity to review the priorities set at the first forum. Specific plans have been made for workshops with partner organisations once the data is available, and we believe that the availability of concrete data will facilitate development of research questions where a more open-ended approach failed. We believe that this process demonstrated the very active engagement of local partners in setting their own research agenda and hope that this can lay the groundwork for international partners and funders to be more intentionally collaborative with local partners – in fact to be led by them – in their research processes even prior to the development of their research questions.

## Data Availability

All data generated or analysed during this study are included in this published article or are available upon request from the authors.
